# Innovative Solid-Phase Extraction Strategies for Improving the Advanced Chromatographic Determination of Drugs in Challenging Biological Samples

**DOI:** 10.3390/molecules29102278

**Published:** 2024-05-12

**Authors:** Mohammadreza Mahdavijalal, Carmine Petio, Giovanni Staffilano, Roberto Mandrioli, Michele Protti

**Affiliations:** 1 Research Group of Pharmaco-Toxicological Analysis (PTA Lab), Department of Pharmacy and Biotechnology (FaBiT), Alma Mater Studiorum—University of Bologna, 40126 Bologna, Italy; mohamma.mahdavijalal@unibo.it (M.M.); michele.protti2@unibo.it (M.P.); 2 Psychiatric Diagnosis and Care Services, Local Health Unit Company (AUSL) of Bologna—IRCCS St. Orsola-Malpighi, 40138 Bologna, Italy; carmine.petio@ausl.bologna.it; 3 Cardiology and Intensive Care Unit, Local Health Company (ASL) of Teramo, 64100 Teramo, Italy; staffilano@gmail.com; 4 Department for Life Quality Studies (QuVi), Alma Mater Studiorum—University of Bologna, 47921 Rimini, Italy

**Keywords:** therapeutic drug monitoring, human biological fluids, mass spectrometry, smart adsorbents, solid-phase extraction, multiple-stage mass spectrometry

## Abstract

In the past few decades, considerable scientific strides have been made in the subject of drug analysis in human biological samples. However, the risk caused by incorrect drug plasma levels in patients still remains an important concern. This review paper attempts to investigate the advances made over the last ten years in common sample preparation techniques (SPT) for biological samples based on solid sorbents, including solid-phase extraction (SPE) and solid-phase micro-extraction (SPME), and in particular in the field of molecularly imprinted polymers (MIPs), including non-stimuli-responsive and stimuli-responsive adsorbents. This class of materials is known as ‘smart adsorbents’, exhibiting tailored responses to various stimuli such as magnetic fields, pH, temperature, and light. Details are provided on how these advanced SPT are changing the landscape of modern drug analysis in their coupling with liquid chromatography-mass spectrometry (LC-MS) analytical techniques, a general term that includes high-performance liquid chromatography (HPLC) and ultra-high performance liquid chromatography (UHPLC), as well as any variation of MS, such as tandem (MS/MS), multiple-stage (MS^n^), and high-resolution (HRMS) mass spectrometry. Some notes are also provided on coupling with less-performing techniques, such as high-performance liquid chromatography with ultraviolet (HPLC-UV) and diode array detection (HPLC-DAD) detection. Finally, we provide a general review of the difficulties and benefits of the proposed approaches and the future prospects of this research area.

## 1. Introduction

The monitoring of pharmaceutical compounds in the biological fluids of patients has been increasingly recognized in recent years as an effective and advantageous measure to enhance the efficacy of drug therapy. Biological matrices include various fluids such as whole blood, serum, plasma, urine, saliva, breast milk, cerebrospinal fluid, and sweat, and tissue samples such as hair, nail, skin, bone, and muscle. Given the close association between circulating drug levels in the bloodstream and their pharmacological effects, the convenience of sampling, and the dynamic fluctuations in drug concentrations detected in plasma and urine, these two matrices are usually considered the most suitable options for the drug analysis process. Due to the presence of a huge number of components in biological matrices and the limited stability of many drugs, obtaining reliable and accurate results from this kind of analysis is a complicated analytical challenge, requiring selective and very efficient sample preparation procedures. This daunting task must anyway be undertaken, since knowing the precise concentration of a medication and its main metabolites in biological tissues makes it possible for physicians to quickly and objectively determine the most effective drug dosage for patients, exploiting known or tentative chemical–clinical correlations (CCC), i.e., relationships between drug/metabolite dose and tissue levels and between tissue levels and therapeutic, unwanted, and toxic effects. This practice, called therapeutic drug monitoring (TDM), is capable of offering clinicians insight into metabolic, bioavailability, toxicity, and pharmacokinetic data [1].

In the last few decades, there has been extensive growth in the field of analytical apparatus for drug evaluation, mainly due to improvements in the wider availability of mass spectrometry (MS) in all of its several types, including high-resolution (HRMS), multiple-stage (MS^n^), and tandem (MS/MS) mass spectrometry. This technological progress has increasingly made it easier to carry out reliable determinations of drugs in complex matrix samples, even at trace and ultra-trace concentrations. However, the effective tracking of drugs remains challenging owing to the existence of matrix interferences in biofluids such as proteins, carbohydrates, lipids, nucleic acids, and metabolites [2]. Technical innovations have not yet made sample preparation techniques (SPT) useless or irrelevant. Thus, an intelligent choice of the correct approach for biological fluid SPT, in conjunction with a proper measuring tool for drug analysis, is of the utmost significance in this field of study. In this regard, solid-adsorbent-based techniques, mainly solid-phase extraction (SPE), and its recent developments, such as dispersive solid-phase extraction (d-SPE), solid-phase microextraction (SPME), and SPE on molecularly imprinted polymers (SPE-MIP), are highly favored and extensively employed [3,4,5,6]. Nevertheless, the development, optimization, and application of effective SPE protocols to be applied to the desired drugs are generally viewed as a taxing and complicated task. Such problems have motivated the development of novel strategies for removing unwanted chemicals from biological samples [4].

The SPE approach often entails the use of relatively large amounts of solvents, raising environmental concerns about solvent toxicity, waste management, and energy usage. In this context, the advancement of miniaturized SPE technology has led to significant shifts in pharmaceutical analysis and offered greener and more ecologically friendly alternatives to traditional SPE procedures, according to the current tenets of Green Analytical Chemistry (GAC) [7] in general and of Green Sample Preparation (GSP) in particular [8,9]. SPME, MIPs, micro-extraction by packed sorbent (MEPS), and volumetric absorptive microsampling (VAMS) are examples of widely used miniaturized SPE approaches that have received a lot of attention from researchers in the past few years. These techniques have largely addressed environmental concerns in addition to producing significant savings in organic solvent usage. Minimal sample requirements, high analytical sensitivity, shortened extraction duration, compatibility with eco-friendly solvents, and minimal waste generation are the distinctive features of miniaturized SPE methods aligned with green chemistry in drug analysis extraction from complex biosamples [10]. 

Anyway, these techniques employ the same kinds of SPE sorbents as classic non-miniaturized ones; thus, any evolution and progress in the sorbent space can be readily transferred into the miniaturization space, providing better performance to environmentally-friendly approaches. Indeed, an expanding body of literature exists, devoted to investigating the performance increase brought about by the use of new SPE adsorbents, and in particular by stimuli-responsive polymers (SRPs). This class of engineered adsorbents possesses an exceptional ability to respond to a specific type of stimulus, such as temperature, pH, and light. In other words, the smart polymer employed in the adsorbent structure exhibits a controlled and reversible alteration in its chemical and physical properties upon exposure to a proper and predetermined stimulus [4]. The unique performance of SRPs enables them to effectively adsorb the desired drug during the extraction process and (in most cases) release it without requiring an extraction solvent in a controlled and intelligent form. The possibility of reusing modified polymeric adsorbents in sample pretreatment operations is another notable feature of these appealing sorbents. Moreover, the smart adsorbents benefit from a high surface-to-volume ratio and minimize the risk of drug loss or degradation during extraction [11]. In this context, using, e.g., magnetic materials as solid sorbents has simplified the SPE process, making it faster and more environmentally friendly than traditional approaches. This technique uses a magnet to facilitate phase separation during the sorbent extraction procedure, eliminating the need for vacuum manifolds or centrifugation processes. Consequently, this approach minimizes the time and energy needed for the extraction operation.

To date, numerous review papers have been published on the synthesis and characterization of adsorbents for use in SPE, d-SPE, SPME, and SPE-MIP approaches for drug extraction from complex biological samples. Hence, this paper does not intend to assess the various methods for synthesizing (smart or non-smart) adsorbents in the presented approaches in scientific papers. Indeed, it aims to foster innovation in the scientific community by illustrating the most significant applications of such advanced adsorbents and how their coupling with increasingly effective and extremely powerful LC-MS analytical techniques is revolutionizing the field of TDM.

## 2. Materials and Methods

### 2.1. Eligibility Criteria 

To achieve the objectives of this review, we investigated research works that focused on quantifying drug extraction from biological fluids using HPLC and GC techniques, with special attention paid to those methods that use MS, MS/MS, MS^n^, and HRMS as the means of detection. Research articles involving the use of other sample preparation and drug analysis systems were excluded, along with in vitro and animal investigations. No gender or age constraints were placed on the subjects (if any) of the considered studies.

### 2.2. Search Strategy 

An organized search was carried out through the following databases: Web of Science, PubMed, Google Scholar, and Scopus. Keywords used in the search included drug extraction, smart solid phase extraction, MIPs, MSPE, SPME, mass spectrometry, LC-MS/MS, and related terms. All the abstracts resulting from the search were screened, and relevant papers were selected and downloaded. Furthermore, a thorough evaluation of references within the relevant articles was carried out in order to discover additional supplemental sources to be incorporated into the study.

## 3. Solid-Phase-Based Extraction Procedures

### 3.1. Application of Non-Stimuli-Responsive Adsorbents

#### 3.1.1. Solid-Phase Extraction (SPE)

Over the past decade, various sorbent materials have been identified and employed for the pre-concentration and extraction of the desired analyte(s) using the SPE technique prior to chromatographic analysis. These adsorbents presented a wide range of choices, from traditional ones such as C_8_, C_18_, and typical phases like silica and alumina to various other sorbents such as hydrophilic-lipophilic balance (HLB), mixed-mode polymeric sorbents (coupling Cx sorbents with cation or anion exchange ones), and more [3]. Likewise, in recent years, a variety of other solid sorbents have been proposed for the preparation of samples, involving magnetic, metal-organic, carbon particles, and polymer-based particles such as MIPs, poly(glycidyl methacrylate-divinylbenzene) (PGM-DVB), and polystyrene-divinylbenzene (PS-DVB) [3]. 

Wojnicz et al. [12] proposed an efficient method for measuring aripiprazole and its metabolite, dehydroaripiprazole, in plasma samples via LC-MS/MS. The suggested approach extracts analytes from 200 µL of plasma sample using a three-step micro-elution SPE with Oasis PRiME HLB 96-well elution plates and isotopically labeled aripiprazole as an internal standard (IS). The PRiME HLB sorbent is an innovative polymeric material that is water-wettable and does not require conditioning or equilibration, thus greatly simplifying extraction procedures. It is reported that the offered approach can eliminate over 99% of primary plasma phospholipids, surpassing alternative successful approaches such as the protein precipitation technique (PPT) or liquid-liquid extraction (LLE). Furthermore, this strategy has demonstrated greater extraction recovery, varying from 96% to 106%, in comparison with previously employed methods in this area. The authors utilized a triple quadrupole MS (QqQ) analyzer with electrospray ionization (ESI) and improved multiple reaction monitoring (SRM) parameters for accurate mass measurement. This optimization involved adjusting the nebulizer pressure to 60 psi, retaining the desolvation gas (nitrogen) at 350 °C, and a flow rate of 12 mL/min [12]. The method offers some green benefits, like sample volume reduction and less chemical waste generation due to the effective removal of phospholipid. Additionally, the elimination of phospholipids from plasma samples using a three-step micro-elution SPE method decreases matrix effects and improves the reliability of the analytical method. Nevertheless, the three-step approach may require more time and effort than simpler procedures such as PPT.

In another paper, Tang et al. [13] introduced an efficient approach using LC-MS/MS to quantify morphine and clonidine in human plasma samples for neonatal pharmacokinetic analysis. In this method, the process of SPE has been performed using a polymeric reversed-phase sorbent, Strata-X, yielding purer sample extracts than other methods like PPT. The method requires a minimum volume of 100 µL of patient plasma, which is valuable for newborns with limited blood samples. The approach revealed a level of accuracy and precision of about 15% for each analyte. The morphine and clonidine measuring limits were 1 to 1000 ng/mL and 0.25 to 100 ng/mL, respectively. Notably, the extraction yields indicated acceptable effectiveness, with values ranging from around 79% to nearly 94%. The authors’ proposed method displays an elevated degree of accuracy and sensitivity through SPE sample preparation. The strategy employed purer sample extracts and eliminated interference more effectively than alternative extraction methods. Further, the suggested approach conserves valuable sample resources, decreases the need for additional sample collection, and lowers anxiety among patients. The application of Strata-X sorbent in this research is consistent with GSP principles as it minimizes the consumption of organic solvents and waste creation, therefore supporting an eco-friendly analytical method. Nevertheless, considering the volume of samples and specific requirements, the use of Strata-X sorbents may hinder the simultaneous handling of samples, thereby reducing experimental capacity and technique effectiveness. Table 1 lists some applications of SPE in drug extraction from biosamples over the last ten years [13].

#### 3.1.2. Dispersive Solid-Phase Extraction (d-SPE) 

In 2003, Anastassiades et al. introduced, a fast, simple, and highly efficient cleanup d-SPE method, to the scientific community [22]. In the d-SPE, the absorbent particles are dispersed throughout the sample matrix rather than being tightly packed into any kind of device, such as an SPE cartridge, column, or disk [23]. This form of dispersion enhances interactions between the sorbent and target analytes, reducing both extraction time and solvent consumption and supporting the development of environmentally friendly and economical methods. After the drug loading process onto the dispersed sorbents, the next important phase is separation, which involves isolating and purifying the solution containing the target analytes through techniques such as centrifugation or filtration. The extracted analytes are ultimately determined following their elution process from the adsorbent. The approach, also known as dispersive micro-solid phase extraction (D-μ-SPE), utilizes small quantities (milligrams) of sorbent. Qian et al. [24] proposed a fast and extremely precise approach for reliable colchicine measurement in plasma and urine. The method involves a combination of in-syringe d-SPE and LC-QqQ with ESI (d-SPE-ESI-LC-MS/MS) in MRM mode, demonstrating very good linearity and accuracy at therapeutic drug levels. The in-syringe dispersive d-SPE approach effectively optimized the quantities of primary secondary amine (PSA) and magnesium sulfate (MgSO_4_). The proposed method demonstrated a linearity range of 0.04–20 ng/mL and limits of detection (LOD) and quantitation (LOQ) of 0.06 ng/mL and 0.2 ng/mL for colchicine in plasma and urine. Mean extraction yields at three different spiking levels in plasma and urine are quite reproducible (93.9–102.68%), with acceptable relative standard deviation (RSD) values [24]. Moreover, the investigation thoroughly validates the analytical approach by assessing matrix effect, stability, dilution integrity, and carryover. The methodology improves analytical precision by removing impurities through the use of in-syringe d-SPE. Additionally, it uses less solvent than traditional extraction techniques, making it more environmentally friendly. The method, despite the success in sample preparation facilitated by DSPE, involves a complex process necessitating broad optimization, thereby elevating the time and complexity requisite for method development.

Marzi Khosrowshahi et al. [25] developed an improved d-SPE technique employing microcrystalline cellulose (MCC) for the precise detection of metoprolol in plasma and wastewater using HPLC-MS/MS. Employing MCC as a sorbent in the d-SPE method provides a green, economically viable, and environmentally friendly alternative. In addition, zinc sulfate (ZnSO_4_) is recommended for precipitating proteins in this study. The paper deals with some experimental parameters such as the optimization of MCC value, salt addition, adsorption time, and elution solvent volume. The proposed strategy indicated reasonable relative recovery rates in the range of 84–93% for plasma and 92–96% for wastewater, with minor effects caused by sample matrices on analyte measurements [25]. The green achievement of this study is the utilization of MCC, a renewable and biodegradable sorbent, as an eco-friendly substitute for conventional extraction sorbents in the d-SPE process of metoprolol from complicated samples. Furthermore, the suggested technique uses a minimal volume of plasma and wastewater with no extra treatment, which reduces sample treatments and resource usage. The avoidance of sample dilution, the avoidance of cartridge clogging, and the usage of minimal plasma volume are some of the advantages of the offered method in the study. In return, the necessity to optimize extraction parameters for other biosample matrices seems a possible barrier to the suggested method, complicating the process of both development and operation. Table 2 presents a brief overview of the application of d-SPE for extracting drugs from human biological samples. 

#### 3.1.3. Solid-Phase Microextraction (SPME) 

SPME is an efficient extraction technique allowing for the direct extraction of the target analyte(s) from an aqueous matrix onto a coated micro-diameter fiber with no need for any solvent. The mechanism of SPME is dependent on the establishment of a partition equilibrium between the analytes present in the sample or headspace and a coated fused silica fiber. The biosample is typically placed in a vial with a septum-type top, and the fiber is either exposed to the sample headspace (HD-SPME) or directly immersed (DI-SPME) in liquid samples. Non-volatile analytes are typically extracted by directly immersing the fiber into the sample, whereas volatile analytes are extracted by placing the fiber directly above the liquid sample in a sealed container for the process of headspace extraction. This approach facilitates and improves the extraction process by enabling the target analyte to transfer directly from the sample matrix to the polymeric stationary phase present on the fiber’s surface. The amount of analyte extracted onto the fiber surface could be affected by the polarity and thickness of the stationary phase coating, the duration of the extraction process, and the analyte concentration in the sample. Moreover, some effective parameters, such as the addition of salt to the sample, agitation, and pH or temperature change, could all be used to improve the efficacy of analyte extraction. Arthur and Pawliszyn [30] pioneered this extraction method in the early 1990s, and it quickly became popular due to its ease of use, rapidity, and effectiveness in addressing drawbacks associated with traditional sample preparation methods, such as multiple preparation steps, high solvent consumption, time-consuming processes, and analyte loss.

Looby et al. [31] developed a high-throughput SPME procedure combined with LC-MS/MS for the determination of tranexamic acid (TXA) in plasma and urine samples. The authors employed a thin-film microextraction (TFME) brush along with an HLB coating for its affinity with TXA and other polar compounds. The suggested strategy demonstrated a 12-fold advancement in the entire procedure of preparing samples, needing just 25 min to treat 96 samples. Overall, the method produced LOQ values of 10 μg/mL and 25 μg/mL for plasma and urine, respectively, with accuracy ranging from 103% to 105% and precision RSD values lower than 8% [31]. This study offers various benefits, including achieving an LOQ of 10 μg/mL for plasma and 25 μg/mL for urine, showing sensitivity for detecting TXA concentrations. The method simplifies sample handling by removing pretreatment processes like derivatization and sample clean-up, optimizing the procedure, and minimizing potential sources of errors. Furthermore, the technique contributes to green methods for analytical chemistry by reducing the requirement for solvent-assisted extraction, intensive sample pretreatment, or clean-up procedures. Despite the ease and improvement that SPME technology brings to drug extraction methods, it may confront difficulties in providing reliable results with the complex chemical compositions in diverse biosamples. Hence, it is important to recognize its potential limitations and investigate complementary procedures that guarantee the reliability and precision of analytical results.

Moreover, Hasegawa et al. [32] assessed the use of MonoTip C_18_ tips for micropipette tip-based SPE combined with GC-MS in order to quantify dextromethorphan in human plasma samples. The authors utilized MonoTip C_18_ tips with bonded monolithic silica gel for sequential extraction and elution with methanol, followed by GC-MS analysis employing a fused silica capillary column and selected ion monitoring (SIM) in positive electron ionization (EI+) mode with a single quadrupole setup. The approach demonstrated an overall 87.4% recovery, excellent linearity in the 2.5–320 ng/mL concentration range, and an LOD of 1.25 ng/mL in plasma [32]. The paper’s green achievement is attributed to its reduced organic solvent usage, minimized waste generation, and faster extraction time compared to traditional extraction methods like SPE and LLE. The use of MonoTip C_18_ tips for micropipette SPE simplifies the sample preparation process. However, the suggested methodology’s use of MonoTip C_18_ tips increases the possibility of extraction efficiency variations, depending on operator performance and tip quality. Further, achieving ideal extraction conditions requires careful optimization for sufficient recovery and sensitivity, which may require significant time and resources. Some instances of SPME employed for drug extraction from biological materials in recent years are listed in Table 3.

#### 3.1.4. Molecularly Imprinted Polymers (MIPs) 

Nowadays, there is a growing trend in academic research to increase the use of MIPs as stationary phases in the drug extraction process, since these smart adsorbents display the capacity to successfully selectively isolate and pre-concentrate the compounds of interest from complex sample matrices [33]. The MIP material is produced through the polymerization of functional and cross-linking monomers surrounding a template molecule, yielding a polymer with a dense three-dimensional cross-linked network. The monomers are selected based on their potential to form non-covalent interactions with the functional groups present in the template molecule. Following the completion of the polymeric stage, the template molecule is separated from the adsorbent, triggering the creation of MIPs, which are characterized by the presence of specific empty space binding sites capable of strongly interacting with the analyte(s) [33,34,35]. 

Combes et al. [36] developed and synthesized two MIPs capable of extracting carbamazepine (CBZ), oxcarbazepine (OXC), and its metabolites from urine. Moreover, the authors compared the efficacy of MIPs versus a non-imprinted polymer (NIP) and a different solid sorbent (Oasis HLB) in extracting the target drugs from urine, emphasizing good selectivity, efficiency, and matrix effect performance. MIP data included extraction recoveries higher than 82% at a 20 ng/mL spike level. Additionally, the LOD value for MIPs combined with LC-MS ranged from 1 to 7 ng/mL [36]. This work represents an efficient starting point for employing MIPs in clinical and TDM analysis in the near future.

The recommended strategy has various advantages, including MIPs’ selective extraction capabilities, which exhibit excellent selectivity in extracting CBZ, OXC, and metabolites from urine samples, even at low levels of concentration. In addition, in contrast to common Oasis HLB sorbents, synthesized MIPs provide higher performance in clean-up efficiency in human urine samples, showing better sample preparation capabilities within analytical workflows. While MIPs provide advantages such as selectivity and accuracy, evaluating their cost-effectiveness against performance standards and comparing them with other extraction methods is essential for establishing their practical value.

Zhou et al. [37] presented an analytical method for measuring vancomycin in plasma samples, utilizing the combination of surface molecularly imprinted solid-phase extraction (SMISPE) and LC-MS/MS. The authors developed SMISPE with teicoplanin as the virtual template and silica gel as the carrier, yielding greater selectivity and quick mass transfer. The resultant SMI was then packed into an SPE cartridge to perform the sample pretreatment process. The approach produced accurate calibration in plasma in the 1–100 ng/mL range. The recovery values varied from 94.3% to 104.0%, with precision RSD consistently less than 10.5%, proving the method’s accuracy and reliability [37]. The method suggested has a high sensitivity, allowing for the detection of vancomycin at concentrations as low as 0.5 ng/mL, guaranteeing precise drug level monitoring. Additionally, the method comes with a fast sample preparation time (15 min), which improves analysis efficiency and convenience. However, integrating SMISPE with LC-MS/MS may require specific equipment, thereby increasing the complexity and cost of analysis. Table 3 reports some important applications of MIPs in the extraction of drugs from human biological samples published over the last ten years.

**Table 3:** Some examples of SPME and MIPs applications in drug extraction from human biological samples over the past ten years.

Analyte(s)	Matrices	Extraction Conditions	Analysis Conditions	Analysis Method	LOD (ng/mL)	Ref.
Dextromethorphan	Plasma	Sorbent: micropipette tip-based C_18_; Extraction Solvent: MeOH; Sample Volume: 100 μL	Column: Equity-5 fused silica capillary column (30 × 0.32, 0.5 μm); Column Temperature: 20 °C for 1 min; then at 20 °C/min to 270 °C; and finally increased at 30 °C/min to 300 °C; Carrier Gas: Helium; Flow rate: 2 mL/min	SPME- GC/MS	1.25	[32]
Beta-blocker drugs	Urine	Sorbent: MIP-SPE (Hydrophilic Co-Monomers: 2-hydroxyethyl methacrylate and glycerol dimethacrylate); Functional Monomer: Methacrylic acid; Crosslinker: Ethylene glycol dimethacrylate; Extraction Solvent: ACN	Column: Shim-Pack XR-ODS C_18_ (100 × 3 mm, 2.2 μm); MP: (0.01%) FA/MeOH (30:70 *v*/*v*) and NH_4_FA (pH 5.0); Detector: MS/MS with ESI-POS; Operating mode: MRM	MIPs-SPE- HPLC- MS/MS	1.0	[38]
Cocaine, its metabolites	Plasma	Sorbent: Synthesized MIPs using ethylene dimethacrylate (monomer); divinylbenzene-80 (cross-linker); Extraction Solvent: dichloromethane; 2-propanol, and ammonium hydroxide (76:20:4)	Column: Phenomenex Kinetex 5 C_18_ (100 × 2.1 mm, 5.0 μm); MP: (A)NH_4_OAc (2 mM)/MeOH; (B)NH_4_OAc (2 mM)/Water; Flow rate: 0.2 mL/min; Detector: ABSciex 3200 Q TRAP LC-MS/MS system with ESI; Operating mode: MRM	MIPs-µ-SPE- HPLC- MS/MS	0.061–0.87	[39]
Roxithromycin	Plasma	Sorbent: polyethylene tablet-shape (magnetic MIP-SPME); Extraction Solvent: MeOH/Acetic acid (5%); Sample Volume: 500 μL	Column: Symmetry C_18_ (150 × 4.6 mm × 5 µm); MP: Acetic acid (0.1%)/(10 mmol/L) NH_4_OAc and MeOH (30:70, *v*/*v*); Flow Rate: 1.0 mL/min, (30 °C); Injection Volume: 20 μL; Detector: MS/MS with ESI-POS; Operating mode: MRM	MMIP- SPME –HPLC- MS/MS	3.8	[40]
Methadone	Plasma	Sorbent: polyethylene tablet-shape (MIP-sol-gel); Extraction Solvent: MeOH; (5 mM) NH_4_FA (pH 4.0) (8:1:1, *v*/*v*/*v*); Sample Volume: 200 μL	Column: Zorbax Bonus-RP (100 × 2.1 mm, 3.5 µm); MP: (0.1%) FA in ACN/water (5:95) and (0.1%) FA in MeOH (45 °C); Detector: MS/MS with ESI-POS; Operating mode: MRM	MIPs-µ-SPE- HPLC- MS/MS	1.0	[41]
Amphetamine	Urine	Sorbent: MIP-gel form; Extraction Solvent: MeOH; Sample Volume: 1000 µL	Column: Zorbax Bonus-RP (100 × 2.1 mm, 3.5 μm); MP: (0.1 mol/L) NH_4_FA in Water and (0.1%) FA in MeOH; Column temperature: 30 °C; Detector: MS/MS with ESI-POS; Operating mode: MRM	MIPs-µ-SPE- HPLC- MS/MS	1.0	[42]
Cannabinoids	Urine	Sorbent: polypropylene porous membrane protected μ-SPE system; Extraction Solvent: Heptane/2-Propanol/Ammonium hydroxide (75:20:5 *v*/*v*/*v*)	Column: RP Kinetex C_18_(100 × 2.1 mm, 2.6 μm); MP: (0.1%) FA in water and (0.1%) FA in meOH; Flow Rate: 0.40 mL/min; Injection Volume: 20 μL; Detector: MS/MS with ESI-POS; Operating mode: MRM	MIPs-µ-SPE- UHPLC- MS/MS	0.032–0.75	[43]
Carbamazepine	Urine	Sorbent: MIPs (55 mg); Cartridge volume: 3000 µL; Extraction Solvent: MeOH	Column: Varian C_18_ Omnispher (150 × 2.1 mm × 5 μm); MP: MeOH/ACN/H_2_O (38:20:42); Flow rate: 0.2 mL/min, (35 °C); Injection Volume: 5 μL; Detector: TQMS with ESI-POS; Operating mode: MRM	MIPs-SPME-LC-MS/MS	1.0	[36]
Cannabinoids	Urine	Sorbent: MIP-MEPS device; Extraction Solvent: ACN; Sample Volume: 500 μL	Column: Poroshell 120 EC-C_18_ (100 × 2.1mm, 2.7 μm); MP: Water + ACN (with 0.1% FA); Flow rate: 0. mL/min (40 °C); Injection Volume: 10 μL; Detector: MS/MS with ESI-POS; Operating mode: MRM	MIPs-SPME-LC-MS/MS	-	[44]
Tranexamic acid	Plasma Urine	Sorbent: HLB-coated SPME; Extraction Solvent: MeOH/Water (50:50 *v*/*v*); plasma: MeOH/ACN/Water (3:3:4); urine: Water/MeOH(90:10); Sample Volume: 1000 µL	Column: (10 × 2.1 mm, 3 µm); Discovery HS F5 guard column(2 ×2.1 mm, 3 μm); MP: Water (99.9%) in 0.1% FA/99.9% ACN (99.9%) in 0.1% FA; Injection Volume: 10 μL	SPME-LC-MS/MS	0.6	[31]
Vancomycin	Plasma	Sorbent: surface molecularly imprinted polymer adsorbent (SMIP) (60 mg); Extraction Solvent: MeOH (5%)	Column: Phenomenex Kinetex Biphenyl (50 × 2.1 mm, 5 μm); MP: (0.1%) FA in ACN + (0.1%) FA in Water; Flow rate: 0.4 mL/min; Injection Volume: 10 μL; Detector: MS/MS with ESI-POS; Operating mode: MRM	MIPs-SPME-LC-MS/MS	1.0	[37]

SPE—Solid-phase Extraction; SPME—Solid-phase Microextraction; MIP—Molecularly Imprinted Polymer; MEPS—Micro-Extraction by Packed Sorbent; ACN—Acetonitrile; MeOH—Methanol; MP—Mobile phase; ESI—electrospray ionization; MRM—Multiple reactions monitoring; NH_4_OAc—ammonium acetate; RP—Reversed phase; HLB—Hydrophilic-Lipophilic Balance; FA—Formic acid; MMIP—Magnetic Molecularly Imprinted Polymer; µ-SPE—Micro-Solid-phase Extraction; NH_4_FA—Ammonium formate.

#### 3.1.5. Microsampling Methods 

In the early 1960s, microsampling was introduced by Dr. Robert Guthrie using the method of dried blood spotting (DBS) for global newborn screening [45]. Within this approach, a small blood volume from a finger- or heel-prick is sampled on a paper card for storage. After drying, discs of suitable diameter are punched out of the DBS card and immersed in a solvent for direct extraction, analysis, or further sample preparation. One of the most important drawbacks of DBS is the hematocrit (Hct) bias. Hct is the fraction of blood volume filled by blood cells, which influences blood viscosity and dispersion on the DBS filter paper. The uneven distribution of blood on the card could affect extraction recovery and the matrix effect. The blood viscosity variability means that drops with different volumes are produced and that different blood volumes are extracted when punching out a constant spot diameter. 

Within the context of bio-sample treatment advances, volumetric absorptive microsampling (VAMS) has acquired prominence in this area by efficiently collecting constant volumes of blood (10, 20, or 30 µL) via a porous, calibrated hydrophilic tip. To date, several studies have confirmed the potential of VAMS in bio-fluid microsampling, achieving an actual Hct independence of sampled volume and thus better reproducibility and quantitative applications [46,47,48]. Like all dried microsampling techniques, VAMS usually provides higher levels of analyte stability than standard wet plasma samples, making it possible to perform room temperature transport and storage in most cases. Furthermore, the VAMS system is easily adapted to at-home blood collection with no need for healthcare experts, making it very convenient for chronic drug monitoring, for patients living far from healthcare facilities, and for patients with reduced mobility. Arguably, determining accurate drug quantities within such a low collected blood volume (≤30 μL) requires the use of advanced, very sensitive, and selective analytical techniques, such as LC-MS/MS. A literature search has revealed that only a limited number of studies on the subject of drug analysis employing the VAMS-LC-MS/MS technique have been published (Table 4). 

**Figure 1:**
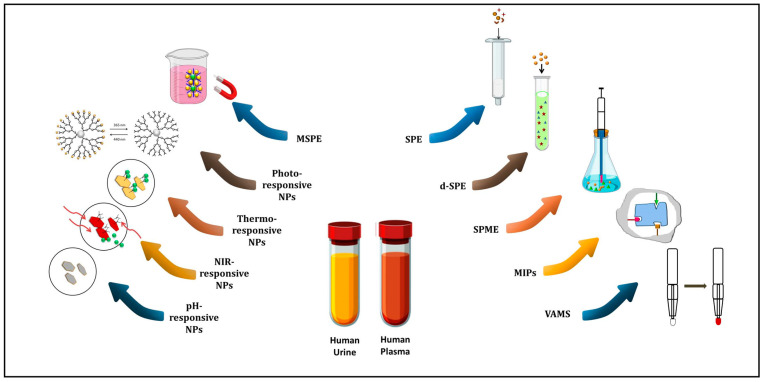
Schematic illustration of biological sample preparation methods for drug analysis before the chromatographic techniques discussed in this review paper.

Paniagua-González et al. [49] created and validated a quick UPLC-MS/MS analytical technique for measuring mycophenolic acid (MPA), tacrolimus (TAC), sirolimus (SIR), everolimus (EVE), and cyclosporin A (CsA) in whole blood (WB). The approach achieved a 2.2 min chromatographic run time through the use of a unique atmospheric pressure interface, UniSprayTM, and VAMS equipment. In this study, the authors highlight the benefits of using UniSprayTM as an ionization source rather than ESI in LC-MS/MS analysis for immunosuppressant medicines, potentially increasing both accuracy and sensitivity in the measurement procedure. The essential difference between these two ionization sources is the application of high voltage: ESI provides it to the spray capillary tip, whereas UniSprayTM directs it to a stainless-steel cylindrical target rod (impactor pin). This interface acts like ESI, but due to the Coandă effect (i.e., the tendency of fluids to flow following the profile of close-by surfaces), the downstream gas flow from the nebulizer follows the curvature of the target rod surface, resulting in smaller droplets and improved analyte desolvation. The authors also exploited statistical research (using Passing–Bablok regression, the intraclass correlation coefficient (ICC), and Bland–Altman plots) as part of the VAMS analytical technique validation. They compared the concentrations of TAC (n = 53) and MPA (n = 20) in liquid venous blood to the levels observed after peripheral blood sampling by fingerprick and VAMS. In this case, the results obtained seem to indicate that lower TAC levels are found in peripheral VAMS compared to venous blood, requiring further study and examination. The recommended method obtained an LLOQ of 0.5 ng/mL for TAC, EVE, and SIR, 20 ng/mL for CsA, and 75 ng/mL for MPA. In addition, the extraction protocol achieved satisfactory recovery rates (≥73.8%) across various Hct levels (0.2–0.6) as well as good robustness and accuracy, ensuring method reliability in a wide variety of clinical situations [49]. The advantage reported in this research is the validation of VAMS as an accurate representation of blood drug concentrations, which are quite similar to those obtained from standard liquid venous blood samples. This validation ensures the method’s integrity, enabling confident exploration of correlations between venous and capillary blood using VAMS. A schematic representation of the sample preparation approaches used prior to the chromatographic techniques covered in this review paper is shown in Figure 1.

**Table 4:** Some examples of VAMS applications in drug extraction from human biological samples over the past ten years.

Analyte(s)	Matrices	Extraction Conditions	Analysis Conditions	Analysis Method	LOD (ng/mL)	Ref.
Piperacillin-Tazobactam, Meropenem, Linezolid, and Ceftazidime	Plasma	VAMS samples (MITRA^®^) with10 μL device, dipped in whole blood, air-dried for 1 h, and stored at −20 °C. DBS with 30 μL whole blood on filter paper, air-dried similarly, and stored at −20 °C with desiccant in a zip-closure plastic bag	Column: Kinetex C_18_ column (100 × 4.6 mm; 2.6 µm), MP: (0.1%) FA in water and ACN, Injection volume:10 µL (40 °C), Detector: MS/MS with ESI-POS, Operating mode: MRM	VAMS- HPLC- MS/MS	-	[50]
Sertraline, Fluoxetine, Citalopram, Vortioxetine, and their metabolites	Whole Blood, Oral Fluid	Microextraction by Packed Sorbent (MEPS) on C_2_ Sorbent; Extraction Solvent: MeOH	Column: Waters XBridge BEH C_18_ column (150 × 2.1 mm, 3.5 μm); MP: Aqueous phosphate buffer(33 mM, pH 3.0)+ 0.3% TEA (solvent A)/ACN (solvent B); Flow rate: 1.0 mL/min (25 °C); Injection Volume: 20 L, Detector: UV-FL, Sertaline, norsertraline, and vortioxetine by UV at at 225 nm, Fluoxetine, Citalopram, norfluoxetine, N-desmethylcitalopram, and N,N-desmethylcitalopram were by FL at λ_em_ = 235 nm, λ_ex_ = 300 nm	VAMS- HPLC- UV-FL	0.3–3.0	[46]
Cefepime	Blood	VAMS™ devices in a 96-well plate	Column: Phenomenex Lux Cellulose-3 column (100 × 4.6 mm, 2.6 µm); MP: NH_4_OAc in water (5 mM, pH 5)(solution A); NH_4_OAc in ACN(5 mM, 0:10 (*v*/*v*))(solution B); Injection volume: 3.0 µL; Flow rate: 0.5 mL/min (25 °C); Detector: MS/MS with ESI-POS; Operating mode: SRM	VAMS- HPLC- MS/MS	-	[51]
Cocaine and its metabolites	Blood or Plasma	Mitra^®^VAMS Microsamplers with polypropylene handle and polymeric material tip; Sample Voluome: 100 µL	Column: XBridge BEH C_8_ column (50 × 3.0 mm; 2.5 µm); MP: (0.5%) FA in water and (0.5%) FA in ACN; Injection volume: 10 µL (25 °C); Flow rate: 0.3 mL/min; Detector: MS/MS with ESI-POS; Operating mode: MRM	VAMS- HPLC- MS/MS	0.3–0.8	[52]
Clenbuterol	Urine	Mitra^®^VAMS Microsamplers with polypropylene handle and polymeric material tip; Sample Voluome: 100 µL	Column: Phenomenex Lux Cellulose-3 column (150 × 3.0 mm, 3 µm), MP: (0.5%) FA in water (pH 7.2) with 1 M carbonate solution and CAN (80:20); Injection volume: 10 µL (25 °C); Detector: MS/MS with ESI-POS, Operating mode: MRM	VAMS- StAGE- LC- MS/MS	0.1	[47]

VAMS—volumetric absorptive microsampling; StAGE—stop-and-go extraction; ACN—Acetonitrile; MeOH—Methanol; MP—Mobile phase; TEA—Triethylamine; ESI—electrospray ionization; MRM—Multiple reactions monitoring; SRM—Selective Reaction Monitoring; NH_4_OAc—ammonium acetate; FA—Formic acid.

### 3.2. Application of Stimuli-Responsive Polymeric Adsorbents

#### 3.2.1. Magnetic Responsive Adsorbents

Magnetic Solid Phase Extraction (MSPE) represents an efficient technique that combines magnetic nanoparticles (MNPs) with classic SPE techniques to separate and extract the target molecule from complex mixtures using a magnetic field. In this frame, the core of MSPE consists of non-magnetic sorbents with magnetic inorganic elements serving as adsorption agents [53]. These MNPs are modified with ligands or coatings tailored specifically for the analyte, guaranteeing selectivity and affinity throughout the extraction process. The MSPE procedure begins by mixing MNPs with the material, similar to d-SPE. This initial stage increases the interaction between the analytes and the MNPs, making the adsorption process a greater success. Upon exposure to an external magnetic field, the magnetically responsive MNPs, along with the adsorbed analytes, are swiftly concentrated and separated. 

Perhaps the most significant benefit of MSPE is that this technique avoids the steps frequently used in SPE procedures, like material packing, vacuum application, centrifugation, or filtration. Moreover, MSPE is extremely adaptable and usually exhibits remarkable selectivity, even when it is applied to complicated matrixes from environmental or biological sources. Since many substances in sample matrixes exhibit diamagnetic behavior, they are incapable of restricting the mobility of magnetic particles during the separation process. The magnetically active center of the adsorbent particles generally includes cobalt, nickel, iron, or their respective oxides. Among these, magnetite (Fe_3_O_4_) is the most frequently employed component [54]. The MSPE technique minimizes analysis time by simplifying the extraction steps and facilitating the simultaneous separation and concentration of analytes. Moreover, the easy separation of the drug-loaded adsorbent with an external magnetic field minimizes the usage of organic solvents, which is consistent with green chemistry principles and avoids harmful waste generation [10]. It is worth mentioning that MSPE approach poses some serious concerns, including the possibility of magnetic NP agglomeration, differences in extraction yields between drugs, and time-consuming sample preparation processes. Overcoming these issues requires cautious attention during method execution [53]. 

Cai et al. reported the successful extraction of several antidepressants (venlafaxine, paroxetine, fluoxetine, norfluoxetine, and sertraline) from plasma and urine samples using magnetic C_18_-Fe_3_O_4_@SiO_2_ NPs and measuring them using UHPLC-MS/MS [55]. The research employed ESI in positive mode at 5500 V in addition to MRM to track specific precursor-to-product ion transitions. Influential factors such as collision energy (from 31 to 65 V), source temperature (500 °C), and declustering potential (from 28 to 65 V) were optimized. The adsorbent produced displayed a rapid state of adsorption/desorption equilibrium during the drug extraction process, with a low amount of the adsorbent (20 mg). The authors achieved satisfactory drug recoveries from plasma and urine samples (77.0% to 119.4%). Additionally, the coefficient of variation (CV%) for all spiked samples fell within an acceptable range of 10.5% [55]. The authors claimed that the proposed C_18_-functionalized MNPs in this study are environmentally friendly, simple to use, and cost-effective and have a higher throughput than traditional liquid-phase extraction and SPE methods for clinical sample pretreatment. The suggested sorbent quickly obtains adsorption/desorption equilibrium in analyte extraction with just 20 mg utilized, and it is reusable for a maximum of ten cycles. However, the MSPE method offered demands more sample preparation time compared to conventional techniques. 

In another study, Heidari and colleagues [56] developed and optimized an MSPE method for the measurement of antihypertensive drugs such as losartan, carvedilol, and amlodipine besylate in plasma samples via HPLC-UV. The authors enhanced the stability of the magnetic Fe_3_O_4_ NPs through a carbon-coating process using a hydrothermal reaction with glucose. They found out that the relative recoveries for carvedilol (93.86–91.67%) and amlodipine besylate (96.81–95.47%) were appropriate, whereas for losartan (63.94–62.11%) it was rather low. They attributed this result to the high binding rate of losartan to plasma proteins. However, the synthesized C/Fe_3_O_4_ MSPE demonstrated remarkable potential by effectively adsorbing target drugs in complex plasma samples without any requirement for a protein precipitation operation [56]. The synthesis of carbon-coated Fe_3_O_4_ nanoparticles, which adheres to green chemistry principles, stands out as a crucial finding of the study. The authors believe that the proposed methodology based on C/Fe_3_O_4_ MNPs represents a pioneering effort in the extraction of losartan, carvedilol, and amlodipine besylate from plasma samples without the necessity of the PPT. Table 5 summarizes the approaches outlined above, along with several other recent developments in the field of drug extraction from biosamples through MSPE for simple evaluation and comparison.

#### 3.2.2. Common Stimuli-Responsive Adsorbents 

Thermo-responsive adsorbents are primarily designed and manufactured by adding thermo-sensitive polymers as functional monomers to the NPs structure. Ideally, temperature changes induce corresponding changes in the adsorbent’s structure, such as expansion, contraction, phase alterations, or surface property alterations, which may be either reversible or irreversible [72]. The synthesis of a thermosensitive polymeric network on the surface of SPE adsorbents not only improves drug loading capacity on the sorbent but also provides the possibility of controlled release of the loaded analyte from biological solutions in a determined temperature range. 

Thermo-responsive polymers feature a distinctive critical solution temperature (CST) that is classified based on the type of monomer utilized as either a lower CST (LCST) or an upper CST (UCST) [72]. The designed drug adsorbents containing polymers with LCST polymers exhibit hydrophilic properties at temperatures lower than their LCST values. The ingress of water into these types of polymers results in their swelling state. On the contrary, when the temperature exceeds the LCST value, these polymers assume a globular conformation and exhibit hydrophobic behavior. Meanwhile, adsorbents made from UCST polymers demonstrate a swollen state when exposed to temperatures exceeding their respective UCST. 

In this regard, poly (N-isopropylacrylamide), or PNIPAM, demonstrates a reversible phase change at its LCST, which takes place at about 32 °C in aqueous solutions [73]. This characteristic enables the possibility of drug release control, allowing the adsorbent to retain the analyte(s) at room temperature and subsequently release it at modestly higher temperatures. Several other polymers, such as poly(ethylene glycol) (PEG), poly(vinyl methyl ether) (PVME), poly(N-vinylcaprolactam) (PNVCL), and poly(vinylpyrrolidone) (PVP), exhibit thermo-sensitive behavior with a low LCST, making them suitable for applications like drug delivery, sensors, and smart adsorbents [63]. It is important to note that some drawbacks, such as the high potential toxicity of thermoresponsive polymers and the high molecular weights of the smart polymer carrier, may hinder their wide adoption in standardized routine drug analysis [74].

Kazemi and colleagues have successfully engineered a thermo-sensitive MIP adsorbent for the accurate quantification of imatinib mesylate in plasma samples [75]. The polymerization procedure was performed by using two separate components: N,N′-methylenebisacrylamide as a cross-linking agent and PNVCL as a temperature-sensitive polymer. The authors evaluated the temperature impact on the synthesized MIPs network (30 to 50 °C) and observed the highest adsorption of imatinib mesylate at 35 °C, which was attributed to the expanded chains of the temperature-sensitive polymer. In contrast, the temperature-dependent release profile indicated a considerable release of imatinib mesylate from the adsorbent at 50 °C. It was believed that elevating the temperature could reduce the size of the cavities of synthesized MIPs and release the loaded drug. They achieved considerable extraction recoveries (90–95%) from plasma and urine with reasonable relative standard deviations (≤0.98%). The technique showed a relatively LOD (1.4 ng/mL), indicating its high efficiency and sensitivity [66]. 

The proposed MIP adsorbent in this paper features temperature-controlled selectivity and benefits from a proper sample preparation time. Moreover, the suggested strategy is environmentally friendly and uses fewer organic solvents and reagents than traditional extraction methods. Nevertheless, the MIP described in this paper requires precise temperature regulation to function reliably. Variable environmental temperatures may present adaptation challenges, compromising the dependability and reproducibility of extraction results. Based on the existing literature, only a handful of studies have reported on the use of stimuli-responsive nano-adsorbents for drug extraction from biological fluids, which are listed in Table 6.

#### 3.2.3. Dual- and Multi-Stimuli-Responsive Adsorbents 

**Figure 2:**
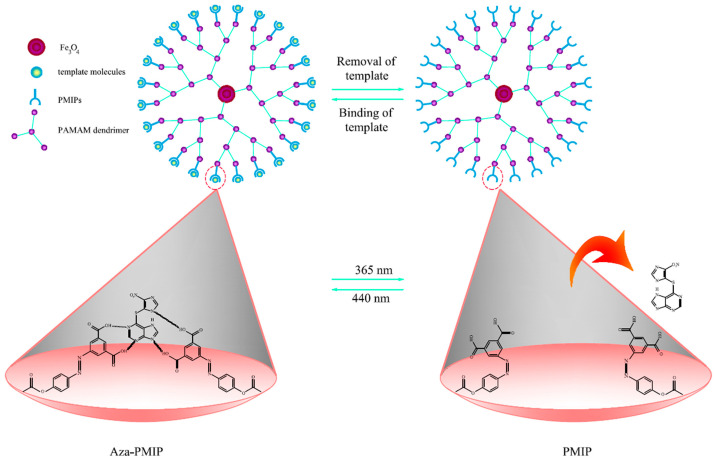
Photo- responsive MIP process of a dendrimer based on MNPs. From H.S. Alaei et al. [89], with permission.

**Figure 3:**
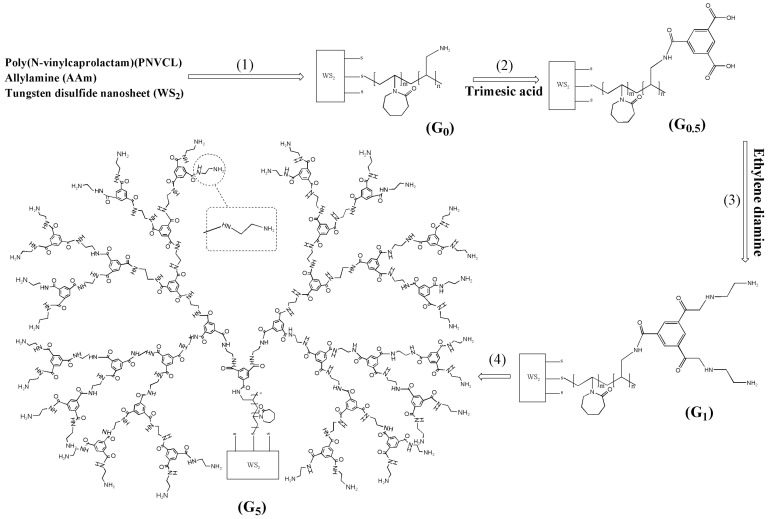
Near-Infrared Irradiation- and Thermo-sensitive adsorbent based on Modified WS_2_ Nano-sheets with five generations of polymeric dendrimers. From M. Mahdavijalal et al. [90], with permission.

**Figure 4:**
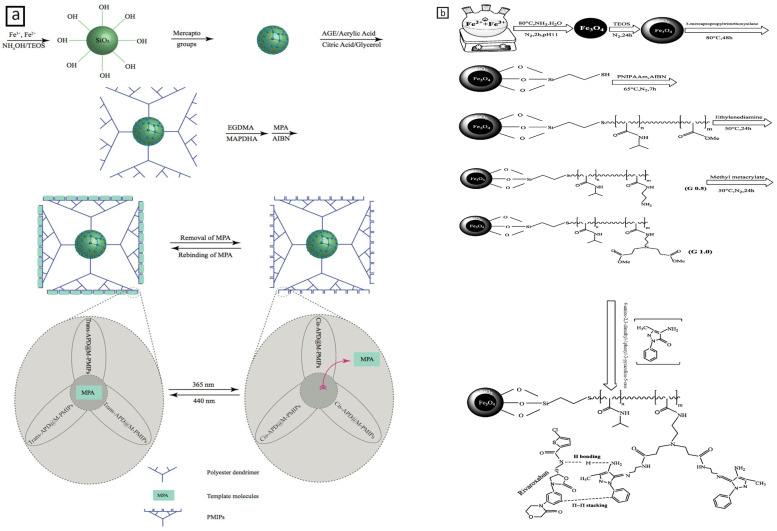
(**a**) A Photo-responsive polymer with MNPs and a polyester dendrimer, from N. Baimani et al. [91], with permission; (**b**) Dendrimer-based and thermo-responsive MNPs, from N. Parham et al. [92], with permission.

In recent years, the field of biomedical research has experienced promising growth due to the introduction of dual- and multi-stimuli-responsive polymeric NPs. These adsorbents respond to a variety of combined signals, such as pH-temperature, pH-magnetic field, temperature-pH-magnetic field, light-temperature, light-magnetic field, temperature-magnetic field, etc., enabling them to adapt to challenging extraction situations (Figure 2, Figure 3 and Figure 4) [81]. The latest important advances in dual- and multi-stimuli-responsive polymeric sorbents for drug analysis applications, with particular focus on their structure and extraction yield performance, are listed in Table 7.

Naghibi et al. developed a modified Fe_3_O_4_ adsorbent employing a thermo-sensitive polymer for the purpose of extracting and quantifying cefexime in biological samples [82]. In the present inquiry, the process of the co-polymer grafting of PNVCL and 3-allyloxy-1,2-propanediol as a thermo-sensitive agent was executed on the surface of Fe_3_O_4_ NPs. They observed that approximately 70% of the incubated cefexime was effectively adsorbed onto the synthesized adsorbent. The adsorbent demonstrated a reasonable rate of extraction recovery for cefexime from both plasma and urine samples, ranging from 71 to 89%. Moreover, the authors observed that the proportion of cumulative cefexime release at 37 °C was considerably greater compared to what occurred at 25 °C. They linked their findings to the deformation of the PNVCL chain on the surface of the adsorbent particles, which changes from a sharp coil to a globule shape when temperature exceeds the LCST limit [82]. 

The dual nanoadsorbent presented by the authors provides advantages including high sensitivity for low-concentration drug detection, fast extraction, and reusability. The magnetic adsorbent minimizes the need for organic solvents, promoting eco-friendly extraction based on green principles. Moreover, the thermosensitive polymer facilitates the process of controlled drug release, offers on-demand release profiles tailored to intended therapeutic needs, and ensures adsorbent reusuability for frequent drug loading and release cycles. However, limitations in drug loading capacity, complexity, longer synthesizing time, and the high cost of nanoparticle production pose challenges to the broader application of this smart drug extraction technique from biological materials.

Taghvimi et al. developed and characterized a novel pH-responsive magnetic NP as an efficient smart adsorbent for the extraction of amphetamine from human urine samples [83]. In this case, block copolymer poly (ethylene glycol)-b-poly(N,N-dimethyl aminoethylmethacrylate-co-maleic acid) was used as a pH-sensitive block copolymer for stabilizing and coating a magnetic adsorbent. The results indicated excellent adsorbent dispersion and drug extraction in urine samples up to 4 mL in volume. The authors obtained a considerable improvement in extraction efficiency with an elevation of pH from 4 to 10. The researchers attributed this observation to the increase in ionization of carboxylic acid groups on the pH-responsive magnetic NPs, followed by an increase in the negative charge density of the NPs, which facilitates the interaction of positively charged amphetamines with the negatively charged sites on the NPs. The HPLC-UV analysis in the study exhibited a 99.84% recovery rate of amphetamine from urine samples [83].

The pH sensitivity of the offered adsorbent in this study optimizes the drug adsorption efficiency and leads to higher extraction yields. Moreover, the magnetic response ability of the adsorbent enables the fast separation of the adsorbent–drug combination from the biological samples, minimizing extraction time. Moreover, the proposed strategy contributes to waste reduction by allowing for efficient extraction with smaller sample amounts. The ability to regenerate and reuse nano-adsorbents for numerous extraction cycles decreases resource consumption, which aligns with the principles of sustainable chemistry. In return, the pH sensitivity of the drug adsorbent could restrict the range of pH situations appropriate for the extraction procedure, reducing its applicability for particular medicines or biological fluids. Furthermore, reusing a pH-sensitive adsorbent could be difficult, demanding harsh conditions or affecting its efficiency over several uses.

Light-sensitive adsorbents enable the precise and remote execution of the drug extraction process from biofluids without resorting to chemicals and with exactly determined timing and location. This type of adsorbent changes its molecular structure when exposed to electromagnetic radiation at specific wavelengths and intensities. These changes can affect the adsorbent’s electrical properties and allow for the manipulation of parameters like size, wettability, and form using light. Typically, this change is reversible and typically occurs in the UV–Vis spectrum range [84]. Recently, numerous light-sensitive molecules were researched for the development of light-responsive materials. These molecules include inorganic compounds such as metal oxides and sulfides (such as ZnS, TiO_2_, MoS_2_, and WS_2_) [85], as well as organic compounds such as azobenzene, stilbene, spiropyran, coumarin, diarylethene, and cinnamate [86,87,88]. Azobenzene undergoes a transformation from trans- to cis-configuration when exposed to UV light at 365 nm. Visible light (at 445 nm) or heat can reverse the process [86]. 

Spiropyran undergoes a photo-initiated isomerization process in response to UV radiation, which changes its hydrophobic ring-closed structure from colorless to a colored hydrophilic ring-opened merocyanine configuration [87,88]. In other words, the structure and dipole moment of the molecule change during the photoisomerization procedure. The twisted structure of spiropyran, consisting of two heterocyclic units, undergoes conversion to a planar extended pi-conjugated merocyanine form that exhibits both zwitterionic and quinoid resonance structures [87]. The spiropyran molecule’s dipole moment shifts greatly from 4.3 D to 17.7 D when the zwitterionic merocyanine structure is formed (Figure 1). This structure can go back to its spiropyran form when exposed to visible light.

As shown in Table 6, only a few investigations have been published in the literature on the use of photo-responsive adsorbents and SPE techniques to extract medicines from biological materials. In addition, the majority of reported experiments have used azobenzene derivatives to extract drugs using photosensitive adsorbents. Alaei and colleagues have developed photo-responsive MIPs for the selective separation of the immunosuppressant azathioprine [89]. The authors synthesized a smart dendrimer-magnetic-based adsorbent utilizing the template of azathioprine, water-soluble 5-[(4-(methacryloyloxy) phenyl)diazenyl] isophthalic acid, and ethylene glycol dimethacrylate. The findings of the research indicate that the rates of recovery of spiking azathioprine from human blood serum (95.85%) and urine (102.71%) have been quite acceptable. Furthermore, a limited recovery of azathioprine (24–35%) from urine and blood samples without UV-Vis (365 nm) irradiation substantially supported the efficacy of the recommended strategy for azathioprine recovery (Figure 2) [89]. 

Generally, photo-responsive adsorbents offer several advantages over traditional ones, including precise and remote control over drug extraction without the need for chemical agents. Additionally, they provide high sensitivity for detecting low drug concentrations in complicated matrices. Through their selective targeting of the analyte of interest and controlled reaction to light, photo-responsive adsorbents can minimize matrix impact. However, challenges exist in optimizing NPs design, ensuring biocompatibility and safety and choosing suitable light sources and parameters. Further research is needed to overcome these challenges and unlock the full potential of these types of smart adsorbents for drug extraction processes from human biological fluids. With continued advancements, these adsorbents hold significant promise for advancing drug analysis and personalized medicine applications.

**Table 7:** Some examples of dual and multi-stimuli responsive nano-adsorbents in drug extraction from human biological samples over the past ten years.

Analyte(s)	Matrices	Extraction Conditions	Stimulus	Analysis Conditions	Analysis Method	Recovery (%)	Ref.
Celecoxib	Plasma Urine	Sorbent: Synthesized Fe_3_O_4_ NPs modified with PNVCL and allylimidazole (NVC/AI-MNP)	Magnetic field, Temperature	Column: C_18_ (15 × 4.6 mm, 5 μm); MP: phosphoric acid, TEA, Water, ACN; Flow Rate: 1.0 mL/min (25 °C); Injection volume: 10 μL; Detector: UV (at 268 nm)	MSPE LC-UV	16–96	[93]
Cefexime	Plasma Urine	Sorbent: Synthesized Fe_3_O_4_ NPs, grafted to PNVCL and 3-allyloxy-1,2-propanediol (Fe_3_O_4_@PNVCL/AP)	Magnetic field, Temperature	Column: Extend-C_18_ column (15 × 4.6 mm, 5 μm), MP: Tetrabutylammonium hydroxide/ACN(3:1 *v*/*v*) (pH 6.5), Flow Rate: 1.0 mL/min (40 °C), Injection volume: 10 μL, Detector: DAD (at 254 nm)	MSPE-HPLC-DAD	71–89	[82]
Amphetamine	Urine	Sorbent: Polymeric magnetic-pH-responsive (block copolymer (Poly ethylene glycol-b-poly (N,N dimethyl amino ethyl methacrylate-co-maleic acid) NPs	Magnetic field, pH	Column: C_18_ column (25 × 4.6 mm, 5 μm); MP: ACN/phosphate buffer (10 mM, pH 3.5)(15/85 (*v*/*v*)); Flow rate: 1.0 mL/min; Injection Volume: 40 μL; Detector: UV	MIPs- HPLC-UV	99.84	[83]
Rivaroxaban	Plasma Urine	Sorbent: magnetic core includes Fe_3_O_4_ and TEOS with modified surface including PNIPAAm	Magnetic field, Temperature	Column: C_18_, MP: (A) 5 mL acetic acid in 1000 mL water, (B): 70 mL ACN in 30 mL mobile A; Flow Rate: 1.0 mL/min (25 °C); Injection volume: 20 μL; Detector: DAD (at 250 nm)	MSPE-HPLC-DAD	11.3–92.5	[92]
Azathioprine	Plasma Urine	Sorbent: dendrimer-coated Fe_3_O_4_ NPs grafted to MIPs matrix, including 5-[(4-(methacryloyloxy)phenyl) diazenyl] isophthalic acid, and ethylene glycol dimethacrylate(dMNPs@PMIPs)	Magnetic field, Light	Column: C_18_ (30 × 3.9 mm, 10 μm); MP: Sodium 1-heptanesulfonate (1.6 g, pH 3.5)/MeOH (70:30, *v*/*v*); Flow Rate: 0.8 mL/min (40 °C); Injection volume: 20 μL; Detector: UV (at 286 nm)	MMIPs- d-SPE HPLC-UV	95.85–102.71	[89]
Methyl prednisolone acetate	Plasma Urine	Sorbent:polyester dendrimer-grafted, photo-responsive MIPs including 5-[(4, 3-(methacryloyloxy) phenyl) diazenyl] dihydroxy aniline and ethylene glycol dimethacrylate	Light	Column: Waters Corporation silica L_3_ (250 × 4.6 mm); MP: n-butyl chloride, Water-saturated n-butyl chloride, tetrahydrofuran, MeOH, and Glacial acetic acid (95:95:14:7:6); Flow Rate:1.0 mL/min; Injection volume: 10 μ; Detector: UV (254 nm)	MIPs- HPLC-UV	96.8–104.2	[91]
Imatinib mesylate	Plasma Urine	Sorbent: Synthesized Fe_3_O_4_ NPs, coated with PNVCL and grafted with chitosan, exhibit dual sensitivity to temperature and pH (Fe_3_O_4_@PNVCL-COOH)	Magnetic field, Temperature, pH	Column: BEH C_18_ (100 × 2.1 mm, 1.7 μm), BEH C_18_ VanGuard pre-column (5 × 2.1 mm, 1.7 μm), MP: FA(0.1%) in Water/MeOH, injection volume: 10 μL(4 °C), Detector: MS/MS with ESI-POS, Operating mode: MRM	MSPE-UPLC-MS/MS	80–91	[94]
Bicaltumide	Plasma Urine	Sorbent: NIR- and Thermo-sensitive NPs composed of WS_2_ nano-sheets and five generation of polymeric dendrimers	Temperature, NIR laser	Column: L1 (10 × 4 mm, 3 μm); MP: TFA (0.01%) in water/TFA (0.01%) in ACN (52:48); Flow Rate: 1.0 mL/min (25 °C); Injection volume: 10 μL; Detector: UV (at 270 nm)	SPE-HPLC-UV	92.12–94.54	[90]
Bicaltumide	Plasma Urine	Sorbent: Grafting of polymer chains including PNVCL, Allylamine, Allyl acetoacetate onto WS_2_ NPs (SMNA)	Temperature, NIR laser	Column: L1 (10 × 4 mm, 3 μm); MP:TFA (0.01%) in water/TFA (0.01%) in ACN (52:48); Flow Rate: 1.0 mL/min (25 °C); Injection volume:10 μ; Detector: UV (at 270 nm)	SPE-HPLC-UV	92.08–94.17	[95]

NPs—Nanoparticles; MSPE—Magnetic Solid-phase Extraction; MIPs—Molecularly Imprinted Polymers; PNIPAAm—Poly(N-isopropylacrylamide), ACN—Acetonitrile; MeOH—Methanol; MP—Mobile phase; TEA—Triethylamine; ESI—electrospray ionization; MRM—Multiple reactions monitoring; FA—Formic acid; PNVCL—poly(N-vinylcaprolactam); AP—3-allyloxy-1,2-propanediol; TEOS—Tetraethyl orthosilicate; SMNA—Smart Modified Nano-adsorbent; TFA—Trifluoroacetic acid; NVC—*N*-vinylcaprolactam; AI—Allylimidazole; MNP—Magnetic nano-particles; MMIPs—Magnetic Molecularly Imprinted Polymers; d-SPE—dispersive Solid-phase Extraction; NIR—Near-Infrared Radiation.

## 4. Conclusions

This review explores recent developments in the field of biological sample preparation through SPE, d-SPE, SPME, and MIP techniques prior to chromatographic techniques coupled with various detectors, paying particular attention to LC and GC methods coupled to any of the several flavors of MS detection. Moreover, new adsorbents with the ability to react to different stimuli are included. In this regard, some action mechanisms, such as magnetic, thermo-responsive, pH-responsive, and photo-responsive polymers, were described in detail as efficient tools in the smart adsorbent structure. The approaches evaluated broadly demonstrated well-suited selectivity, controlled release and extraction processes, a suitable level of sensitivity, and easy sample preparation procedures. Advancements in drug analysis largely facilitate medical treatments, but it is important to recognize and tackle ongoing obstacles. Designing efficient NPs (size, shape, and surface) and optimizing the extraction parameters (including pH, temperature, and flow rates in particular for “smart” sorbents) are crucial aspects for the efficient execution of these techniques. This review has also highlighted the key place LC-MS has occupied in the last few years in the TDM of pharmacological drugs, thanks to its superior selectivity and sensitivity. At the same time, it is also evident that the coupling of “smart” adsorbents of any kind to LC-MS is still relatively uncommon, probably due to the fact that the former are still in their infancy and that further important strides need to be made before they are ready for commercial distribution and routine application. In conclusion, these advancements represent a promising path to improving drug analysis methods, with the final goal of improving patient care through enhanced medication monitoring. It is our opinion that, in the near future, ongoing studies and developments in this field will provide enough knowledge and production improvements to make “smart” adsorbents the ideal pretreatment option for LC-MS applications of TDM, thus providing advanced reliability to these therapeutic practices.

## Figures and Tables

**Table 1:** Some examples of SPE application in drug extraction from human biological samples over the past ten years.

Analyte(s)	Matrices	Extraction Conditions	Analysis Conditions	Analysis Method	LOD (ng/mL)	Ref.
GHB, Ketamine, Norketamine, Phenobarbital, Thiopental, Zolpidem, Zopiclone, Phenytoin	Urine	Sorbent: SPE Strata Screen C_18_ Cartridge/LLE; Sample Volume: 400 μL; Extraction solvent: EA, DE, H (1:1:1, *v*/*v*/*v*); Sorbent: SPE Strata Screen C_18_ Cartridge/LLE; Sample Volume: 400 μL; Extraction solvent: EA, DE, H (1:1:1, *v*/*v*/*v*)	Column: Poroshell C_18_, 100 × 3.0 mm, 2.7 µm; MP: FA, ACN(50:50 *v*/*v*); Flow rate: 0.3 mL/min; Injection volume: 10.0 μL (60 °C); Detector: MS/MS with ESI; operating mode: MRM	SPE-HPLC-MS/MS	0.59–49.5	[14]
Catecholamines, Metanephrines	Urine	Sorbent: SPE Strata-X-CW; Extraction solvent: FA, MeOH (2:98 *v*/*v*); Sample Volume: ≥1000 μL	Column: Unison C_18_ (100 × 2.0 mm, 3 μm); MP: water/FA (99.9:0.1, *v*/*v*) and ACN-FA (99.9:0.1, *v*/*v*); Flow rate: 0.3 mL/min; Injection volume: 1.0 μL (40 °C); Detector: MS/MS with iFunnel Technology source and ESI-POS; Operating mode: MRM	SPE-HPLC-MS/MS	3.5–7.4	[15]
Colistin, Methanesulphonate Colistin	Plasma Urine	Sorbent: SPE Oasis WCX 96-well plate; Sample Volume: 420 μL; Extraction solvent: ACN/water (30/70 *v*/*v*), and (6%) FA	Column: Phonomenex Kinetex XB-C_18_ (100 × 2.1 mm, 2.6 µm); MP: ACN/MeOH (1:1, *v*/*v*) and 0.1% FA in Water; Flow rate: 0.4 mL/min; Injection volume: 5.0 μL (30 °C); Detector: MS/MS with ESI-POS; Operating mode: SRM	SPE- UHPLC- MS/MS	13–25.1	[16]
Paliperidone	Plasma	Sorbent: SPE Oasis PRiME HLB, SPE cartridge (30 mg); Sample Volume: 250 μL	Column: Thermo Betabasic (100 × 4.6 mm, 8.5 μm), MP: MeOH/NH_4_OAc (70:30 *v*/*v*); Flow rate:1.0 mL/min; Detector: MS/MS with ESI-POS; Operating mode: SRM	SPE-HPLC-MS/MS	-	[17]
Aripiprazole, Dehydro-aripiprazole	Plasma	Sorbent: SPE Oasis PRiME HLB 96-well elution plate (3 mg); Sample volume: 200 μL; Extraction solvent: ACN/MeOH/NH_4_FA (5 mM, pH 4.0) (8:1:1, *v*/*v*/*v*)	Column: ACE C_18_-PFP (4.6 × 100 mm, 3 μm); MP: NH_4_FA(5 mM,pH 4.0)/ACN (65:35, *v*/*v*); Flow rate: 0.6 mL/min; Injection Volume:5.0 μL (25 °C); Detector: MS/MS with ESI-POS; Operating mode: MRM	SPE-HPLC- MS/MS	-	[12]
Clonidine, Morphine	Plasma	Sorbent: SPE Strata-X polymeric (30 mg/mL); Extraction solvent: ACN/MeOH (1:1 *v*/*v*); Sample Volume: 150 μL (plasma), 10 μL (urine)	Column: Inertsil ODS_3_ C_18_, (100 × 3 mm, 4 μm); MP: FA (0.1%) in Water + FA (0.1%) in MeOH; Flow rate: 0.2 mL/min; Injection volume:10.0 μL (25 °C); Detector: MS/MS with Turbo Ion Spray; Operating mode: MRM	SPE-HPLC- MS/MS	0.2–0.15	[13]
Aripiprazole, Dehydro-aripiprazole, Olanzapine, Risperidone, Paliperidone, Quetiapine, Clozapine, Caffeine	Plasma	Sorbent: HLB-Oasis PRiME HLB 96-well μElution Plate; Extraction Solvent: ACN with 0.1% FA; Sample Volume: 200 µL	Column: ACE C_18_-PFP (pentafluorophenyl) (100 × 4.6 mm, 3 μm); MP: FA (0.2%)-ACN (pH 3.0) (65:35, *v*/*v*); Flow rate: 0.6 mL/min (25 °C); Injection Volume: 5 μL; Detector: MS/MS with ESI-POS; Operating mode: MRM	µ-SPE- HPLC- MS/MS	-	[18]
Imatinib,Dasatinib, Nilotinib,Bosutinib, Ponatinib,Ruxolitinib, Ibrutinib,Filgotinib, Tofacitinib,Baricitinib, Peficitinib,Caffeine	Plasma	Sorbent: PRiME μ-SPE MCX (mixed-mode cation exchange); Extractin Solvent: 5% NH4OH in MeOH solution (1:1, *v*/*v*) and water	Column: Poroshell EC-C_18_ (75 × 2.1 mm, 2.7 μm); MP: Water/ACN with 0.1% FA (82:12, *v*/*v*); Flow rate: 0.5 mL/min (60 °C); Injection volume: 5 μL; Detector: MS/MS with ESI-POS; Operating mode: dMRM	µ-SPE- HPLC- MS/MS	-	[19]
Palbociclib, Abemaciclib	Plasma	Sorbent: SPE Oasis PRiME HLB; Extraction Solvent: MeOH; Sample Volume: ≥200 µL	Column: Waters CORTECS C_18_ (50 × 4.6 mm, 2.7 μm); MP: NH_4_OAc/Acetic acid (10 mM) and ACN; Flow rate: 0.8 mL/min; Injection volume: 40.0 μL (40 °C); Detector: MS/MS with ESI-POS; Operating mode: SRM	SPE-HPLC-MS/MS	-	[20]
Cefdinir	plasma	Sorbent: Bond Elut Plexa, PCX 30 mg/cc; Extraction Solvent:ACN/MeOH(50:50)	Column: Sigma Aldrich, Kromasil C_18_ with security guard C_18_ (4 × 3 mm); Flow rate: 1.0 mL/min; Injection volume: 15.0 μL (25 °C); MP: ACN/MeOH/Water (0.01% TFA) (45:45:10 *v*/*v*/*v*); Detector: MS/MS with ESI-POS; operating mode: MRM	SPE-HPLC-MS/MS	-	[21]

SPE—Solid-phase Extraction; µ-SPE—Micro Solid-phase Extraction; HLB—Hydrophilic-Lipophilic Balance; ACN—Acetonitrile; MeOH—Methanol; NH_4_FA—Ammonium formate; MP—Mobile phase; GHB—gamma-hydroxybutyrate; FA—Formic acid; EA—ethyl acetate; DE—diethyl ether; H—hexane; LLE—Liquid-liquid Extraction; ESI—electrospray ionization; MRM—Multiple reactions monitoring; dMRM—Dynamic multiple reactions monitoring; SRM—Selective Reaction Monitoring; NH_4_OAc—ammonium acetate; TFA—Trifluoroacetic acid.

**Table 2:** Some examples of d-SPE application in drug extraction from human biological samples over the past ten years.

Analyte(s)	Matrices	Extraction Conditions	Analysis Conditions	Analysis Method	LOD (ng/mL)	Ref.
Bromazepam, Nitrazepam, Lorazepam, Alprazolam, Triazolam, Flunitrazepam, Nimetazepam, Etizolam, Diazepam	Serum Urine	Sorbent: Oasis HLB gel (50 μL) into a micro test tube; Extraction solvent: MeOH	Column: Poroshell 120 EC-C_18_ column (100 × 4.6 mm, 2.7 μm); MP: NH_4_FA (pH 3.0, 10 mM)/ACN (70:30, *v*/*v*); Flow rate: 0.5 mL/min (40 °C); Detector: TOF MS with ESI-POS	d-SPE HPLC–TOF	1–10	[26]
Cyclophosphamide, Cytarabine, Dacarbazine, Doxorubicin, Epirubicin, Etoposide, 5-Fluorouracil, Gemcitabine, Ifosfamide, Methotrexate, Paclitaxel, Vinblastine, Vincristine	Urine	Sorbent: mixture of Oasis HLB^®^ and C_18_; Extraction solvent: H_2_O–MeOH (50:50, *v*/*v*)	Column: Kinetex PFP (100 × 2.10 mm; 2.6 μm); MP: ACN/water and (0.1%) FA; Flow rate: 0.5 mL/min (20 °C); Detector: MS/MS with Turbo ESI source; Operating mode: SRM	d-SPE HPLC–MS/MS	0.01–33.3	[27]
Cocaine, 3,4-Methylenedioxy Methamphetamin	Post mortem blood	Sorbent: Primary Secondary Amine; Extraction Solvent: ACN, EA	Capillary Column: DB-1 ms (30 × 0.25 mm × 0.25 mm); Carrier Gas: Helium, Run Time: 20.5 min; Temperature Ramp: 20 °C/min to 200 °C, 10 °C/min to 300 °C; Run Time: 20.5 min	d-SPE- GC-MS	0.02–0.03	[28]
Amoxicillin,Penicillin, Tylosin artrate, Roxithromycin, Clarithromycin, Azithromycin, Erythromycin,Tetracycline, Chlortetracycline, Ofloxacin, Enrofloxacin,Ciprofloxacin, Terramycin,Norfloxacin, Sulfadiazine, Sulfamethazine, Trimethoprim, Olaquindox,	Urine	Sorbent: roQ™ QuEChERS Dispersed SPE kit; Extraction solvent: MeOH/FA; Sample Volume: 200 μL	Column: RP Gemini NX-C_18_ (50 × 2 mm, 3 μm); MP: Water with (0.1%) FA/MeOH with (0.1%) FA; Flow rate: 0.5 mL/min (31 °C); Injection Volume: 5 μL; Detector: MS/MS with ESI-POS; Operating mode: MRM	d-SPE HPLC–MS/MS	0.11–14.29	[29]
Metoprolol	Plasma	Sorbent: Microcrystalline cellulose (MCC); Extraction Solvent: MeOH	Column: C_18_ (Agilent 15 × 4.6 mm, 5 μm); MP: MeOH/water (25:75, *v*/*v*) and (0.1%) FA; Flow rate: 0.3 mL/min (40 °C); Detector: MS/MS with ESI-POS; Operating mode: MRM	d-SPE HPLC–MS/MS	0.3	[25]
Colchicine	Plasma Urine	Sorbent: Magnesium Sulfate (MgSO_4_) and primary secondary amine (PSA); Extraction Solvent: Water/FA in Water/MeOH/ACN	Column: Waters XBridge™ BEH C_18_ (100 × 2.1 mm, 2.5 μm); MP: Ammonia/MeOH; Flow rate: 0.35 mL/min (30 °C); Detector: MS/MS with ESI-POS; Operating mode: MRM	d-SPE HPLC–MS/MS	0.6	[24]

SPE—Solid-phase Extraction; d-SPE—dispersive Solid-phase Extraction; ACN—Acetonitrile; MeOH—Methanol; NH_4_FA—Ammonium formate; MP—Mobile phase; EA—ethyl acetate; ESI—electrospray ionization; MRM—Multiple reactions monitoring; SRM—Selective Reaction Monitoring; RP—Reversed phase; FA—Formic acid; TOF—Time-of-Flight; HLB—Hydrophilic-Lipophilic Balance.

**Table 5:** Some examples of magnetic nano-adsorbents in drug extraction from human biological samples over the past ten years.

Analyte(s)	Matrices	Extraction Conditions	Stimulus	Analysis Conditions	Analysis Method	Recovery (%)	Ref.
Ephedrine Methamphetamine	Urine	Sorbent: Carbon-coated Fe_3_O_4_ NPs (C/MNPs)	Magnetic field	Column: Analytical C_18_ column (25 × 4.6 mm, 10 μm);MP: ACN/phosphate buffer (10 mM, pH = 3.5); Flow Rate: 1.5 mL/min; Injection Volume: 20 μL	MSPE- HPLC- UV	98.71–97.87	[57]
Phenytoin, Carbamazepine, Diazepam	Plasma Urine	Sorbent: porous magnetic graphene oxide-cyclodextrin polymers (MGO-CDP)	Magnetic field	Column: ODS2 C_18_ (250 × 4.6 mm, 5 μm); Flow Rate: 1.0 mL/min (35 °C); Injection volume: 20 μL; Detector: DAD (230 nm)	MSPE - HPLC- DAD	77.4–87.5 92.4–97.0 6.9–100.9	[58]
Cocaine and its metabolites	Urine	Sorbent: PLS@SMPS@Fe_3_O_4_; Extraction Solvent: MeOH, ACN(4:1, *v*/*v*); Sample Volume: 1000 μL	Magnetic field	Column: XDB C_18_ (4.5 × 150 mm, 5 μm); Guard Column: XDB C_18_, (4.5 × 12.5 mm, 5 μm); MP: (2 mM) NH_4_FA and (0.05%) FA in Water)/(2 mM) NH_4_FA and (0.05%) FA in ACN; Injection Volume: 5 μL, Detector; MS/MS with ESI-POS, Operating mode: MRM	M-d-SPE HPLC–MS	75.1–96.3	[59]
Amitriptyline, Chlorpromazine	Plasma Urine	Sorbent: Magnetite-MCM-41 (Fe_3_O_4_) (15 mg); Extraction Solvent: ACN; Sample Volume:15 mL	Magnetic field	Column: (25 × 0.2 mm, 0.33 μm); Temperature: 220 °C for 3 min, raised to 270 °C at 10 °C/min, then 270 °C for 3 min; Detector: Agilent Technology 5973 inert mass; Carrier Gas: Helium; Flow rate: 1 mL/min	MSPME-GC/MS	0.008–0.01	[60]
Morphine	Plasma Urine	Sorbent: MMIPs composed of Fe_3_O_4_ NPs coated with SiO_2_-NH_2_ (Fe_3_O_4_/SiO_2_-NH_2_)	Magnetic field	Column: XDB-C_18_ column (50 × 4.6 mm,1.8 μm); MP: Acetate buffer (10 mM, pH 4.0, and (0.1% *w*/*v*) 1-octanesulfonic acid sodium) mixed with ACN (60:40, *v*/*v*); Flow Rate: 1.0 mL/min (25 °C); Injection Volume: 20 μL; Detector: Diode array (280 nm)	MMIPs- UHPC- DAD	84.8–105.5 94.9–102.8	[61]
6-mercaptopurine	Plasma	Sorbent: vinyl-modified Fe_3_O_4_ NPs; modified by a silica layer, and functionalized by methacryloxypropyl trimethoxysilane (Fe_3_O_4_@MPS)	Magnetic field	Column: Acquity UPLC BEH shield RP (150 × 2.1mm, 1.7 μm); MP: ACN/FA (0.1%) (85:15 *v*/*v*); Flow Rate: 0.75 mL/min (40 °C); Detector: MS/MS with ESI-POS; Operating mode: MRM	MMIPs- HPLC–MS/MS	85.94–97.62	[62]
Tricyclic, Amitriptyline, Imipramine	Plasma Urine	Sorbent: Combination of Fe_3_O_4_ and TMU-10 (porous shell) for MSPE process (Fe_3_O_4_@TMU-10)	Magnetic field	Column: ODS (250 × 4.6 mm, 5 μm); MP: phosphate buffer (10 mM, pH 4.0) and KClO_4_(25 mM)/ACN(65:35 *v*/*v*); Flow Rate: 1.0 mL/min; Detector: UV (220 nm)	MSPE- HPLC–UV	90.5–99	[63]
Losartan Carvedilol Amlodipine	Plasma	Sorbent: carbon-coated Fe_3_O_4_ magnetic NPs (C/Fe_3_O_4_)	Magnetic field	Column: Eurospher 100–5 C_18_ with precolumn Vertex Plus Column (250 × 4.6mm, 5 μm); MP: MeCN/MeOH/phosphate buffer (25 mM) (36.5:20:43.5); Flow Rate: 0.1 mL/min; Injection Volume: 20 μL, Detector: UV(240nm)	MSPE- HPLC- UV	62.11–96.81	[56]
Carbamazepine	Plasma Urine	Sorbent: Synthesized MMIPs using 4-vinyl pyridine, divinylbenzene, dimethylf ormamide, and coated on magnetic chitosan NPs (Fe_3_O_4_@CS@MIP)	Magnetic field	Column: Hedera ODS-2 (250 × 4.6mm, 5 µm); MP: FA (0.2%)/TEA (0.5%) and organic phase (40%)(MeOH); Flow Rate: 1.0 mL/min (35 °C); Detector: DAD (285 nm)	MMIPs- HPLC–DAD	88.22–101.18	[64]
Venlafaxine Paroxetine Fluoxetine Norfluoxetine Sertraline	Plasma Urine	Sorbent: C_18_-functionalized magnetic silica NPs (C_18_-Fe_3_O_4_@SiO_2_ NPs)	Magnetic field	Column: ZORBAX Eclipse Plus C_18_ (50 × 2.1 mm, 1.8 μm); MP: ACN and (0.1%) FA; Flow Rate: 0.40 mL/min (25 °C); Injection Volume: 2 μL; Detector: MS/MS with ESI-POS; Operating mode: MRM	MSPE- UHPLC–MS/MS	89.1–110.9	[55]
Ibuprofen	Urine	Sorbent: TiO_2_ NPs and C-anofibers modified magnetic Fe_3_O_4_ nanospheres (TiO_2_@Fe_3_O_4_@C-NFs)	Magnetic field	Column: C_18_ (150 × 4.6 mm, 5 μm); MP: ACN/MeOH (50:50 *v*/*v*); Flow Rate: 1.2 mL/min (25 °C); Injection Volume: 20 μL	MSPE HPLC- DAD	97–100	[65]
Morphine,6-onoacetylmorphine,Amphetamine, Methamphetamine, Codeine, Cocaine, Dolantin, Benzoylecgonine	Urine	Sorbent: Graphene oxide–Fe_3_O_4_ nanocomposite (GO–Fe_3_O_4_)	Magnetic field	Column: ACQUITY UPLC BEH C_18_ (100 × 2.1 mm, 1.7 μm); MP: NH_4_FA (10mM) and FA (0.1%) in water/MeOH; Detector: MS/MS with ESI-POS	MSPE- UHPLC-MS/MS	80.4–105.5	[66]
Phenytoin Sodium	Plasma	Sorbent: Synthesized Fe_3_O_4_ NPs with porous structure MIL-101(Cr) shell, and Phe-imprinted polymer (Fe_3_O_4_@MIL-101(Cr)@MIP)	Magnetic field	Column: Kromasil C_18_ (150 × 4.6 mm, 5 μm); MP: Water/MeOH (55:45 (*v*/*v*)); Flow Rate: 1.0 mL/min (30 °C); Injection volume: 20 μL; Detector: UV (220 nm)	MMIPs HPLC–UV	89.1–101	[67]
Fluconazole Voriconazole	Rat Plasma	Sorbent: Modified Fe_3_O_4_NPs with TEOS and APTES and being combined with MOF-5 (Fe_3_O_4_@NH_2_)	Magnetic field	MP: MeOH/Water (60:40, *v*/*v*); Flow Rate: 1.0 mL/min (30 °C); Injection volume: 10 μL; Detector: UV (210 nm)	MSPE- HPLC–UV	86.8–78.6	[68]
Letrozole	Plasma	Sorbent: Acetic Acid-Functionalized Magnetic NPs (AA-FSLN-MNPs)	Magnetic field	Column: RP Intersil column (ODS, octadecylsilane) C_18_ (250 × 4.6 mm, 5 μm); MP: ACN/phosphate buffer (10 mM, pH = 5.5) (50/50 (*v*/*v*)); Flow Rate: 1.5 mL/min (25 °C); Injection Volume: 20 μL; Detector: FL (λ_ex_ = 240 nm, λ_em_ = 296 nm)	MSPE- HPLC-FL	93.5–104	[69]
Amiodarone Lidocaine	Plasma	Sorbent: synthesized Fe_3_O_4_ NPs coated with silica and tetraethyl orthosilicate was used for MIPs (MNP-SMIPs)	Magnetic field	Amiodarone: Column: Perfectsil C4 (150 × 3.9 mm, 5 mm); MP: ACN/phosphate (0.05 M, pH 6); Flow Rate: 1.5 mL/min (25 °C), Detector: UV (240 nm). Lidocaine: Column: Perfectsil C_18_(300 × 3.9mm, 5 mm); MP: ACN/glacial acetic acid (0.9 M, pH 3.4); Flow Rate: 1.5 mL/min (25 °C); Detector: UV (254 nm)	MMIPs HPLC–UV	91.38–97.33	[70]
Paclitaxel	Urine	Sorbent: Synthesized by modifying Fe_3_O_4_ NPs with diamino benzidine tetra chloro hydrate (DABTC-Fe_3_O_4_ NPs)	Magnetic field	Column: RP Inertsil ODS-3 C_18_ (250 × 4.6 mm, 5 μm), MP: ACN/Methyl alcohol (1:1 (*v*/*v*))	MSPE- HPLC–DAD	99–105	[71]

NPs—nanoparticles; MIPs—Molecularly Imprinted Polymers; MSPE—Magnetic Solid-phase Extraction; TEOS—tetraethyl orthosilicate; APTES—3-aminopropyl-triethoxysilane; MOF—Metal-organic framework; PLS—divinyl benzene and vinyl pyrrolidone; SMPS—SiO_2_ and methacrylic acid-3-(trimethoxysilyl) propyl ester; M-d-SPE—Magnetic-dispersive Solid-phase Extraction; ACN—Acetonitrile; MeOH—Methanol; MP—Mobile phase; ESI—electrospray ionization; MRM—Multiple reactions monitoring; RP—Reversed phase; FA—Formic acid.

**Table 6:** Some examples of stimuli-responsive nano-adsorbents for drug extraction from human biological samples over the past ten years.

Analyte(s)	Matrices	Extraction Conditions	Stimulus	Analysis Conditions	Analysis Method	Recovery (%)	Ref.
Triamterene	Plasma Urine	Sorbent: photo-sensitive hollow MIPs, composed of silica micro spheres, a water-soluble azobenzene derivative, ethylene glycol dimethacrylate, and triamterene (PHMIP)	Light	Column: Phenomenex Luna 5u C_18_ (250 × 4.6mm, 5 μm); MP: MeOH/Water (65:35, v:v); Flow Rate:0.4 mL/min; Detector: UV (at 273, and 360 nm)	MIPs- HPLC-UV	93.4–98.5	[76]
Gemcitabine	Serum Urine	Sorbent: Thermo-responsive MIPs made of PNVCL, allylaceto acetate, N,N′-methyl enebisacryl amide, and azobis isobutyronitrile as an initiator (MIP-AA/VC)	Temperature	-	MIPs - HPLC-UV	75–95	[77]
Imatinib mesylate	Plasma Urine	Sorbent: Thermo-sensitive MIPs composed of PNVCL, 1-vinyl-2-pyrrolidone, methyl methacrylate, and N,N′-ethylenebisacrylamide	Temperature	Column: Zorbax Extend C_18_ (15 × 4.6 mm, 3 μm); MP:(A)Sodium 1-octanesulfonate monohydrate in water/ACN and sulfuric acid(7:3 *v*/*v*); (B) Sodium 1-octanesulfonate monohydrate in water/ACN and sulfuric acid(1:9 *v*/*v*); Flow Rate: 2.0 mL/min (25 °C); Injection volume: 50 μL; Detector: UV (260nm)	MIPs - HPLC-UV	90	[75]
Celecoxib	Plasma Urine	Sorbent: Thermo-sensitive MIPs composed of PNVCL, 2-hydroxy ethyl methacrylate, Ethylene glycol dimethacrylate (MIMs)	Temperature	Column: L1 (25 × 4.6 mm, 5 μm); MP: MeOH, ACN, and phosphate buffer (pH = 3) (3:1:6 (*v*/*v*)); Flow Rate: 1.5 mL/min (25 °C); Injection volume: 25 μL; Detector: UV (215 nm)	MIPs - HPLC- UV	93–91	[78]
Antibiotics	Biological Samples	Sorbent: pH-sensitive MIPs composed of 4-vinyl phenyl boronic acid, dimethyl amino ethyl methacrylate, N,N′-methylene bisacrylamide, and ethylene glycol dimethacrylate (tylosin-MIP (D13))	pH	For Tylosin: Column: C_18_ (250 × 4.6mm,5 μm); MP: mono potassium phosphate solution (0.025 mol/L, pH 2.5)/ACN (7:3, *v*/*v*); Flow Rate: 1.0 mL/min (25 °C); Detector: UV (290 nm) For Spiramysin: Column: C18 (250 × 4.6 mm, 5 μm); MP: KH_2_PO_4_-K_2_HPO_4_ buffer (0.0167 mol/L, pH 6.5)/ACN (6:4, *V*/*V*); Flow Rate: 1.0 mL/min (40 °C); Detector: UV (232nm)	MIPs - HPLC-UV	75.6	[79]
Ribavirin	Urine	Sorbent: pH-sensitive MIPs composed of 3-allyloxy-1, 2-propanediol, acrylic acid, Divinylbenzene, N,N′-methylene bisacrylamide (MIP Adsorbent)	pH	-	MIPs - HPLC-UV	102	[80]

MIPs—Molecularly Imprinted Polymers; ACN—Acetonitrile; MeOH—Methanol; MP—Mobile phase; PNVCL—poly(*N*-vinylcaprolactam); MIMs—Molecularly imprinted membranes; PHMIP—photoresponsive hollow molecularly imprinted polymer.
